# Preterm birth: seven-year retrospective study in a single centre population

**DOI:** 10.1186/s13052-019-0643-9

**Published:** 2019-04-11

**Authors:** Roberta Granese, Eloisa Gitto, Gabriella D’Angelo, Raffaele Falsaperla, Giovanni Corsello, Donatella Amadore, Gloria Calagna, Ilaria Fazzolari, Roberta Grasso, Onofrio Triolo

**Affiliations:** 1 Obstetrics and Gynecology Unit, Department of Human Pathology of Adult and Childhood “G. Barresi”, University Hospital “G. Martino”, via Consolare Valeria 1, Gazzi, Messina, Italy; 2 Neonatal and Pediatric Intensive Care Unit, Department of Human Pathology of Adult and Childhood “G. Barresi”, University Hospital “G. Martino”, via Consolare Valeria 1, Gazzi, Messina, Italy; 3 General Pediatrics and Pediatric Acute and Emergency Unit, Policlinico-Vittorio-Emanuele University Hospital, University of Catania, Catania, Italy; 4 Department of Maternal and Child Health, University of Palermo, Palermo, Italy; 5 Obstetrics and Gynecology Unit, “Villa Sofia Cervello Hospital”, University of Palermo, Piazza Salerno 1, Palermo, Italy; 6 Department of Clinical and Experimental Medicine, University of Messina, Messina, Italy

**Keywords:** Birth, Prematurity, Risk factors, Cervical length, Trans-vaginal cervical screening

## Abstract

**Background:**

Preterm birth is a health and social problem, considered the leading cause of neonatal mortality worldwide. It is associated with higher rates of neurodevelopmental morbidity, sensorineural impairments and other complications. The aim of the study was to describe the incidence and the major risk factors associated with preterm birth.

**Methods:**

We performed a single center, observational and retrospective Cohort study in the Division of Obstetrics and Gynaecology, University Hospital “G. Martino”, Messina. Clinical records of all pregnant women who delivered from 1st January 2010 to 31 of December 2016 were collected.

**Results:**

In the 7 years considered, a total of 7954 pregnant women were included in our study. The majority of all preterm births were due to infants born late preterm (71.83%), 26.45% were due to preterm and 1.72% to extremely preterm. The preterm cohort had a higher proportion of history of preterm delivery (*p* < 0.0001), and unmarried (*p* = 0.003) and underweight or obese patients (*p* < 0.0001). In addition, prematurity was associated with presence of uterine anomalies (*p* < 0.0001), vaginal/urinary infections (*p* = 0.02), poli/oligohydramnios (*p* < 0.0001), maternal diabetes (*p* = 0.004), hypertension (p < 0.0001), short cervical length (p < 0.0001).

**Conclusions:**

We suggest prompt identification of all risk factors associated with preterm birth to apply immediate and appropriate specific interventions.

## Background

Preterm birth (PTB) is a serious health and social problem, considered the leading cause of neonatal mortality worldwide [1, 2]. It is associated with higher rates of neurodevelopmental morbidity, sensorineural impairments and other complications of the respiratory, gastrointestinal and renal systems. Epidemiologic evidence also suggests that preterm babies have a significantly increased risk of many chronic degenerative diseases in adulthood, including coronary heart disease, stroke, hypertension, and type II diabetes mellitus [3]. The incidence rates are higher in underdeveloped countries (11.8%) compared to those most developed (9.3%) [4]. The high incidence of this condition even in most developed countries is due to the fact that even though the incidence of preterm delivery is decreasing for greater prevention of known risk factors, it is also increasing due to iatrogenic PTB [5], an improvement of reproductive technology with, consequently, multiple gestations and also an increased maternal age [6].

According to World Health Organization (WHO), preterm birth is defined as any birth before 37 completed weeks of gestation [7]. A decreasing gestational age is associated with increasing complications for premature babies. The gestational age cut off used to distinguish a PTB from spontaneous abortion varies by geographic areas. In our study the cut off considered is 21 weeks and 5 days, as suggested by “Florence Card” in 2008 [8] and reported in the protocol of our Hospital. In the US, for example, 20 weeks is used as the lower gestational age limit [9], while in other regions, especially lower and middle income countries, 28 weeks is often used as the lower limit [9]. We must therefore consider the lower gestational age cut off when we compare PTB rates between different geographic areas.

The research carried out and the preventive measures adopted in this field over the years have allowed a reduction of the incidence of PTB demonstrating the importance of identifying the risk factors associated with this condition [10].

The aim of this study was to assess the trend of the incidence of preterm birth and the major risk factors, combining both maternal risk factors (RFs) and cervical length (CL) and with the inclusion in our cohort of women with a history of previous PTB.

## Methods

We performed a single center, observational and retrospective Cohort study in the Division of Obstetrics and Gynaecology, University Hospital “G. Martino”, Messina, Italy. As a standard protocol, each patient, on admission, signed an informed consent allowing data collection for research purposes. The study design was in accordance with the Helsinki Declaration and was approved by the Institutional Review Board of the University Hospital involved. All the design, analysis, interpretation of data, drafting and revisions followed the guidelines for reporting observational studies (STROBE) [11], available through the EQUATOR (Enhancing the QUAlity and Transparency Of health Research) network (http://www.equatornetwork.org/).

Clinical records of all pregnant women who delivered from 1st January 2010 to 31 of December 2016 were collected, while patients who underwent cerclage, pregnancies complicated by intrauterine fetal death or serious fetal malformation and multiple pregnancies have been excluded (Table 1).

**Table 1:** Clinical and demographic characteristics of control and preterm cases

Characteristics		Controls (*n* = 7315)	Preterm Cases (*n* = 639)	*p*-value
Maternal age (years), mean (SD)		31.87 (6.04)	31.68 (5.65)	0.025
Pre-pregnancy weight (kg), mean (SD)		63.44 (13.98)	63.73 (13.32)	0.2
Maternal BMI, n (%)	Underweight	293 (4)	102 (16)	< 0.0001*
	Normal	4462 (61)	262 (41)	
	Overweight	1682 (23)	51 (8)	
	Obesity	878 (12)	224 (35)	
Marital status, n (%)	Married	5998 (82)	492 (77)	0.003*
	Unmarried	1317 (18)	147 (23)	
Parity	Nulliparous	4096 (56)	367 (57.5)	0.5
	Parous	3219 (44)	272 (42.5)	
Prior history of PTB, Yes/No, n (%)		256/7059 (3.5/96.5)	40/428 (8.5/91.5)	< 0.0001*
Treatment for cervical dysplasia				
Yes/No, n (%)		110/ 7205 (1.5/98.5)	13/626 (2/98)	0.2
Cervical Length (mm), median (IQR)		38 (34–42)	31 (23–37)	< 0.0001*
Uterine Anomalies, Yes/No, n (%)		66/7249 (0.9/99.1)	22/617 (3.4/96.6)	< 0.0001*
Vaginal/Urinary Infections Yes/No, n (%)		1463/5852 (20/80)	115/524 (18/82)	0.02*
Poly/oligohydramnios				
Yes/No, n (%)	No	7096 (97)	518 (81)	< 0.0001*
	Polyhydramnios	37 (0.5)	13 (2)	
	Oligohydramnios	182 (2.5)	102 (9)	
Hypertension, Yes/No, n (%)		366/6949 (5/95)	115/524 (18/82)	< 0.0001*
Thyroid disease, Yes/No, n (%)		1024/6291 (14/86)	83/556 (13/87)	0.5
Diabetes, Yes/No, n (%)		732/6583 (10/90)	93/546 (14.5/85.5)	0.004*
Maternal Surgery, Yes/No, n (%)		2677/4638 (36.6/63.4)	252/387 (39.5/60.5)	0.2
Psychological Disorders, Yes/No, n (%)		37/7278 (0.5/99.5)	7/632 (1.1/98.9)	0.3
Smoking, Yes/No, n (%)		658/6657 (9/91)	51/588 (8/92)	0.5

We defined preterm birth all the deliveries before 36 + 6/7 week/day of gestation according to the WHO [7]. We planned to look at subgroups of PTBs, such as extremely early preterm (i.e. 21 + 5–23 + 6), preterm (i.e. 24–33 + 6) and late preterm (i.e. 34–36 + 6) [12].

### Statistical analysis

Statistical analysis was conducted using SAS version 9.2 (SAS Institute Inc., Cary, NC, USA). Continuous variables are presented as mean (SD) or median (interquartile range), where appropriate, and categorical variables as frequencies and percentages. Differences between control and preterm cases were assessed using unpaired Student’s t-tests or Mann–Whitney U test, as appropriate. The chi-square test was used to compare frequencies. Bivariate associations were tested by Spearman correlation. A conservatively adjusted a priori alpha was set at *p* < .01 due to multiple comparisons.

## Results

**Fig. 1:**
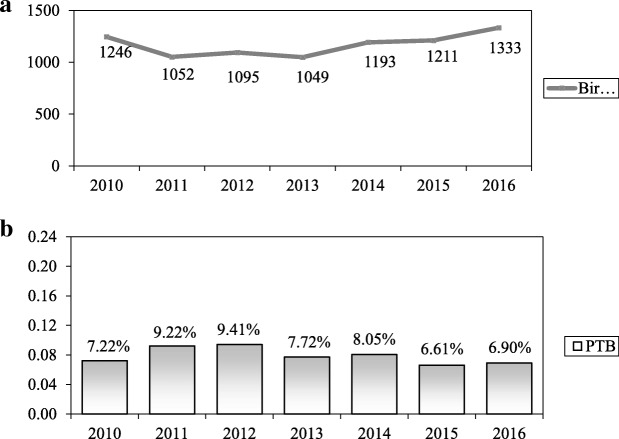
**a** Trends of annual births in our Institution. **b** Trends of preterm birth annual cases in our Institution

A total of 8179 pregnant women were admitted to deliver in our Institution in the period considered [Fig. 1a]. Seven thousand nine hundred fifty-four cases were included in our study and 225 were excluded as they did not fall within the inclusion criteria. The preterm cases were 639 (7.8%). Demographic and clinical characteristics of the preterm cohort and the term controls are shown in Table 1.

The rate of preterm birth in our Institution has shown a relatively stable or a slightly decreasing trend from 7.2 to 6.9% over the 7 year period (Fig. 1b).

**Fig. 2:**
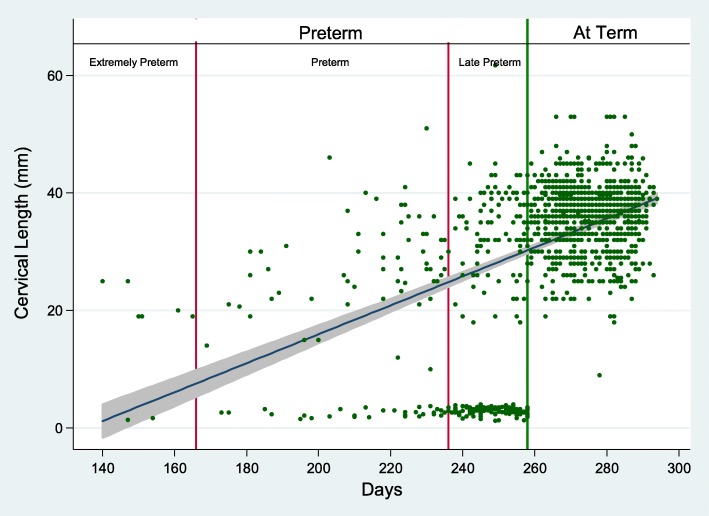
Correlation between cervical length and gestation at birth in preterm cases and controls

The majority of all preterm births were due to infants born late preterm, 71.83% (*n* = 459), 26,45% (*n* = 169) were due to preterm and 1.72% (*n* = 11) to extremely preterm (Fig. 2).

The preterm cohort showed a higher proportion of history of a prior spontaneous PTB (*p* < 0.0001), unmarried women (*p* = 0.003) and underweight or obese patients (p < 0.0001). In addition, PTB was associated with presence of uterine anomalies (*p* < 0.0001), vaginal/urinary infections (*p* = 0.02), poli-oligodramnios (*p* < 0.0001), maternal diabetes (*p* = 0.004) and hypertension (*p* < 0.0001).

The median value of cervical length measured from 16th to 24th week of pregnancy was 31 mm in cases who delivered preterm and 38 mm in controls, demonstrating a significant difference between the two groups (*p* < 0.0001) (Table 1 e Fig. 2). The CL measurement, using a cut off of 25 mm according to the SIEOG guidelines (28), showed a sensibility of 78%, demonstrating to be an adequate screening test for the women at risk of PTB, even if the specificity was only 3%.

Among PTB, cervical length positively correlated with gestational age (r = 0.42, p < 0.0001) (Fig. 2). A sub-analysis of preterm birth categories based on gestational age showed a stronger correlation between gestational age and cervical length among preterm and extremely preterm (r = 0.50, *p* = 0.0002), while no correlation was found among late preterm (r = 0.01, *p* = 0.85). Among PTB, cervical length correlated with older mothers (r = 0.21, *p* = 0.025). No other correlations were found.

## Discussion

In our observational and retrospective Cohort study of pregnant women admitted to an Obstetric Department in Southern Italy, the incidence of preterm birth was 7.8%. The rate of preterm birth decreased from 7.2 to 6.9% (Fig. 1b), together with an increase of births in our Hospital (Fig. 1a). However, the value remains high, considering that preterm birth is associated with most of the pregnancy-related mortality and and short and long-term disability [2].

The slightly decreasing rate of PTB could be, partially, explained with the utmost attention in the prevention of this condition based on the known risk factors. Many features are associated with PTB, such as maternal demographic characteristics [6], nutritional status/ physical activity, prior ob/gyn history, current pregnancy characteristics (i.e. vaginal bleeding, uterine contractions, poly/oligohydramnios, psychological problems, use of drugs or infection) [13–16]. Among these risk factors, history of PTB and short CL are considered the most important predictive factors [17–20]. Particularly, a short cervix in the second trimester (i.e. a cervix measure < 15 mm, in a single pregnancy) [21] is a powerful predictor of spontaneous PTB, regardless of obstetrical history [22].

In our analysis, we only included data on risk factors, which were clearly reported in all clinical records or significantly present in the folders. We report a significant association between PTB and risk factors such as vaginal/urinary infections, underweight or obese patients, unmarried status, the presence of uterine anomalies, poly/oligohydramnios, hypertension, diabetes, a history of PTB and, finally, a short cervical length. Many of these risk factors could be identified with a good medical history and also treated, leading to an improvement of the risk at the beginning of pregnancy.

In our study, particular attention has been given to CL measurement. While “late preterm” cases demonstrated a large variability of CL values, the “preterm” and “very early” preterm cases were more correlated with short cervical length, independently to a history of previous preterm. This data, confirming previous studies [23], assumes that cervical length helps to identify better the case at risk for early and very early PTB. Probably, in late preterm cases, other risk factors should be also considered, such as maternal disease (i.e.: severe hypertension resistant to medical therapy) or fetal pathological conditions which prompt the gynaecologist to induce labor, determining an iatrogenic PTB.

Our findings suggest a universal cervical screening (i.e. not only for women at higher risk) should be performed in order to discover, at an early stage, asymptomatic women with a short cervix (2–5% of total) [24] and to administer a preventive therapy [25, 26]. The CL measurement could be taken during the midtrimester ultrasound screening by all obstetric ultrasonographers who have received appropriate training. Transvaginal ultrasound is safe, and when performed by trained operators results are reproducible with a relatively low inter-observer variation rate of 5–10% [27]. Currently, the cervical measurement is performed only in women with a higher risk of preterm birth or with symptoms of preterm labour (to reduce hospitalization for tocolysis) [28, 29], in women who undergo cervical cerclage or in twin pregnancies [28]. Data on the utility of the cervical length measurement is reported in many papers [23, 25, 28–31] but, according to the guidelines of the Italian Society of Ultrasound in Obstetrics and Gynaecology (SIEOG), the use of ultrasound for the evaluation of the uterine cervix in a low-risk population is not supported by sufficient scientific evidence [28]. This statement has been confirmed in the NICE (National Institute for Health and Care Excellence) guideline [32] and in the SCOG (Society of Obstetricians and Gynaecologists of Canada) guidelines, which do not consider the consequent therapeutic interventions valid [33]. Cervical screening is one tool that can be utilized to identify women at increased risk in order to allow for interventions to prevent, delay, or prepare for preterm births, without the need to undergo to biochemical testing for specific markers for preterm labour. Many authors have questioned if universal midtrimester transvaginal CL screening meets the criteria outlined by the WHO of an adequate screening test [34]. Some authors [35, 36] concluded that this screening for women with a singleton gestation, followed by treatment with vaginal progesterone for those with a short cervix, meets all 10 criteria outlined by the WHO for endorsing the implementation of a screening test in clinical medicine [34].

Also the recent Italian guidelines of the Confalonieri Ragonese Foundation recommend performing a cervical measurement during the second trimester ultrasound screening (19–23.6 weeks) of all pregnant women and to administer 200 mg of Progesterone to those women with a cervix between 10 and 20 mm [21]. This suggestion is motivated by the results of a systematic review where it was shown that Progesterone administration to a sample of women without a history of PTB and with a cervix between 10 and 20 mm demonstrated a significant reduction of PTB and neonatal morbidity, while in the subgroup with a cervical length < 10 mm or between 21 and 25 mm did not give any benefits in prevention of PTB [37]. Another recent systematic review and meta-analysis [26] from randomised controlled trials confirmed that vaginal progesterone was associated with a significant reduction in the risk of preterm birth, respiratory distress syndrome, composite neonatal morbidity and mortality, birth weight < 1500 and < 2500 g and admission to the neonatal intensive care unit. The study reported a reduction of about 40–50% of neonatal death, respiratory distress syndrome, intraventricular haemorrhage and proven neonatal sepsis. Moreover, maternal adverse events, congenital anomalies and adverse neurodevelopmental and health outcomes at 2 years of age did not report any difference between groups, confirming that vaginal progesteron is, at the same time, efficacious and safe [26].

We also use to administrate vaginal progesterone in all the cases at risk for a short CL. This procedure has probably contributed, together with all other preventive measures, to the slight reduction of preterm birth over the years in our Hospital.

## Conclusions

Based on our results, we suggest prompt identification of all risk factors associated with preterm birth to apply immediate and appropriate specific interventions.

We also recommend, confirming the evidence of other studies [26, 38–40], a transvaginal CL measurement during the midtrimester ultrasound screening, in order to identify the women most at risk who could benefit of a progesterone therapy, without the need to undergo to furthers biochemical testing for specific markers for PTB.

All these procedures could reduce the rate of PTB and the associated neonatal morbidity and mortality.

## Abbreviations

CL: Cervical length
NICE: National Institute for Health and Care Excellence
PTB: Preterm birth
RFs: Risk factors
SCOG: Society of Obstetricians and Gynaecologists of Canada
SIEOG: Italian Society of Ultrasound in Obstetrics and Gynaecology
WHO: World Health Organization
